# Novel role of platelet endothelial cell adhesion molecule-1 (PECAM-1) in facilitating TGF-beta-mediated inhibition of T cell function

**DOI:** 10.1186/2051-1426-3-S2-P242

**Published:** 2015-11-04

**Authors:** Debra Newman, Tamara Adams, Vidhyalakshmi Arumugam, Theresa Bluemn, Guoping Fu, Matthew Riese

**Affiliations:** 1 Blood Research Institute, Blood Center of Wisconsin, Milwaukee, WI, USA; 2 Medical College of Wisconsin, Milwaukee, WI, USA

## 

Transforming Growth Factor β (TGFb) is an immunosuppressive cytokine that inhibits pro-inflammatory functions of T cells, and is a major contributor to abrogating T_H_1 and cytotoxic T cell activity against tumors. While canonical signal transduction through effector Smads has been well-defined as a requirement for TGFb-mediated inhibition of T cells, essential non-canonical pathways have not, to date, been defined. This abstract describes the identification of Platelet Endothelial Cell Adhesion Molecule-1 (PECAM-1), CD31, as a novel facilitator of non-canonical TGFβ signal transduction in T cells. Subcutaneously injected tumor cells known to require TGFβ-mediated suppression of immunity for clearance grew more slowly in PECAM-1^-/-^ mice relative to wild type counterparts, and T cells isolated from PECAM-1^-/-^ mice demonstrated relative insensitivity to TGFβ-induced inhibition of IFNγ production and proliferation. Similarly, human T cells lacking PECAM-1 expression demonstrated decreased sensitivity to TGFβ in a manner that could be partially restored by re-expression of PECAM-1. Phosphorylation of PECAM-1 on an Immunoreceptor Tyrosine-based Inhibitory Motif (ITIM) and resultant binding of the inhibitory Src homology 2 domain-containing tyrosine phosphatase-2 (SHP-2) was observed after co-incubation of T cells with TGFβ and anti-CD3, and inducible co-localization of PECAM-1 with the TGFβ receptor complex was identified using co-immunoprecipitation, confocal microscopy and proximity ligation assays. These studies indicate an unexpected role for PECAM-1 in enhancing crucial inhibitory functions of TGFβ in T cells and suggest that targeting of the PECAM-1/TGFβ inhibitory axis represents a novel means to overcome TGFβ-dependent immunosuppression within the tumor microenvironment.

